# Supplementary material for: Spontaneous spinal cord infarctions: a systematic review and pooled analysis protocol
Source: BMJ Open. 2023 Jun 21;13(6):e071044. doi: 10.1136/bmjopen-2022-071044 (PMC10314618; doi:10.1136/bmjopen-2022-071044)
Supplement: Supplementary data [file bmjopen-2022-071044supp002.pdf]

Supplementary file 2: planned search strategy

**Database**	**Search strategy**
PubMed	((idiopathic OR spontaneous OR "non-surgical" OR "nonsurgical" OR "non-iatrogenic" OR "non iatrogenic" OR "noniatrogenic" OR "non-traumatic" OR "nontraumatic") AND ("spinal cord isch*" OR "spinal cord infarct*" OR "spinal isch*" OR "spinal infarct*")) OR ("spinal cord stroke" OR "spinal stroke" OR ”spinal artery infarct*” OR ”spinal artery isch*”) Filters: English
Web of Science	((idiopathic OR spontaneous OR "non-surgical" OR "nonsurgical" OR "non-iatrogenic" OR "non iatrogenic" OR "noniatrogenic" OR "non-traumatic" OR "nontraumatic") AND ("spinal cord isch*" OR "spinal cord infarct*" OR "spinal isch*" OR "spinal infarct*")) OR ("spinal cord stroke" OR "spinal stroke" OR ”spinal artery infarct*” OR ”spinal artery isch*”) (All Fields) and English (Languages)
Embase	((idiopathic OR spontaneous OR 'non-surgical' OR 'nonsurgical' OR 'non-iatrogenic' OR 'non iatrogenic' OR 'noniatrogenic' OR 'non-traumatic' OR 'nontraumatic') AND ('spinal cord isch*' OR 'spinal cord infarct*' OR 'spinal isch*' OR 'spinal infarct*') OR 'spinal cord stroke' OR 'spinal stroke' OR 'spinal artery infarction' OR 'spinal artery ischemia' OR 'spinal artery ischaemia') AND ([article]/lim OR [article in press]/lim OR [data papers]/lim OR [erratum]/lim OR [review]/lim) AND [humans]/lim AND [english]/lim AND [clinical study]/lim
