# Supplementary material for: Spontaneous spinal cord infarctions: a systematic review and pooled analysis protocol
Source: BMJ Open. 2023 Jun 21;13(6):e071044. doi: 10.1136/bmjopen-2022-071044 (PMC10314618; doi:10.1136/bmjopen-2022-071044)
Supplement: Author's manuscript [file bmjopen-2022-071044.draft_revisions.pdf]

BMJ Open

BMJ Open is committed to open peer review. As part of this commitment we make the peer review history of every article we publish publicly available.

When an article is published we post the peer reviewers' comments and the authors' responses online. We also post the versions of the paper that were used during peer review. These are the versions that the peer review comments apply to.

The versions of the paper that follow are the versions that were submitted during the peer review process. They are not the versions of record or the final published versions. They should not be cited or distributed as the published version of this manuscript.

BMJ Open is an open access journal and the full, final, typeset and author-corrected version of record of the manuscript is available on our site with no access controls, subscription charges or pay-per-view fees (http://bmjopen.bmj.com).

If you have any questions on BMJ Open's open peer review process please email info.bmjopen@bmj.com

BMJ Open

Spontaneous spinal cord infarctions: a systematic review and pooled analysis protocol

Journal:	*BMJ Open*
Manuscript ID	bmjopen-2022-071044
Article Type:	Protocol
Date Submitted by the Author:	13-Dec-2022
Complete List of Authors:	El-Hajj, Victor Gabriel; Karolinska University Hospital, Neurosurgery<br>Stenimahitis, Vasilios; Karolinska University Hospital, Department of Neurology; Karolinska Institutet, Department of Clinical Neuroscience<br>Gharios, Maria; Karolinska University Hospital, Department of Neurology<br>Mahdi, Omar Ali; Karolinska University Hospital, Department of Neurology<br>Elmi-Terander, Adrian; Löwenströmska Hospital,<br>Edström, Erik; Karolinska Institutet, Department of Clinical Neuroscience
Keywords:	Stroke < NEUROLOGY, Stroke medicine < INTERNAL MEDICINE, NEUROLOGY

SCHOLARONE™
Manuscripts

Title: Spontaneous spinal cord infarctions: a systematic review and pooled analysis protocol

Author information

Author notes

[1]Victor Gabriel El-Hajj* (victor.gababriel.elhajj@stud.ki.se)

[1,2]Vasilios Stenimahitis*, MD (vasilios.stenimahitis@ki.se)

[1]Maria Gharios, BS (mcg02@mail.aub.edu)

[1]Omar Ali Mahdi (omar.ali.mahdi@stud.ki.se)

[3]Adrian Elmi-Terander, MD, PhD (Adrian.elmi.terander@gmail.com)

[2]Erik Edström, MD, PhD (erik.edstrom.1@ki.se)

*Contributed equally

Affiliations

[1]Department of Neurology, Karolinska University Hospital, Stockholm, Sweden

[2]Department of Clinical Neuroscience, Karolinska Institutet, Stockholm, Sweden

[3]Stockholm Spine Center, Löwenströmska Hospital, Upplands-Väsby, Sweden

Corresponding author

Victor Gabriel El-Hajj

Address: Karolinska University Hospital, Department of Neurosurgery, Eugeniavägen 6, 4th floor, Solna, Stockholm 17164.

ORCID: https://orcid.org/0000-0001-9479-761X

Email: victor.gabriel.elhajj@stud.ki.se

Number: +46765889449

Keywords: Spinal cord infarction, Stroke, Spontaneous infarction, Spinal cord injury

Word count: 1850 (excluding title page, abstract, statements & references).

For peer review only - http://bmjopen.bmj.com/site/about/guidelines.xhtml

Abstract

Introduction

Spinal cord infarction (SCInf) is a rare ischemic event that manifests with acute neurologic deficits. It is typically classified as either spontaneous, defined as SCInf without any inciting event, or periprocedural, which typically occur in conjunction with vascular surgery with aortic manipulations. While periprocedural SCInf have recently been the subject of intensified research, especially focusing on the primary prevention of this complication, spontaneous SCInf remains less studied.

Methods and Analysis

Electronic databases including PubMed, Web of Science and Embase, will be searched using the keywords ”spinal cord”, “infarction”, “ischemia” and “spontaneous”. The search will be set to provide only English studies published from database inception. Editorials, letters, and reviews will also be excluded. Reference lists of relevant records will also be searched. Identified studies will be screened for inclusion, by one reviewer in the first step and then three in the next step to decrease the risk of bias. The synthesis will address several topics of interest including epidemiology, presentation, diagnostics, treatment strategies, outcomes, and predictors. The review aims to gather the body of evidence to summarize the current knowledge on spinal cord infarctions. This will lead to a better understanding of the condition, its risk factors, diagnosis, and management. Moreover, the review will also provide an understanding of the prognosis of patients with SCInf with respect to neurologic function, quality of life, and mortality. Finally, this overview of the literature will allow the identification of knowledge gaps to help guide future research efforts.

Ethics and dissemination

Ethics approval was not required for our review as it is based on existing publications. The final manuscript will be submitted to a peer-reviewed journal.

For peer review only

Strengths and limitations

- Our wide search strategy and limited set of exclusion criteria allow many studies to be included, ensuring adequate coverage of the topic and correct identification of knowledge gaps.
- By providing a comprehensive synthesis of the body of evidence, the data can form the basis for management guidelines and future research efforts.
- We suspect that the quality of data does not suffice to perform a meta-analysis, consequently limiting the level of evidence that can be attained.

For peer review only

For peer review only - http://bmjopen.bmj.com/site/about/guidelines.xhtml

Introduction

Spinal cord infarction (SCInf) is a rare ischemic occurrence representing only a fraction of all ischemic strokes (1%) [1–4] and approximately 6% of all acute myelopathies [5]. The presentation of SCInf is characterized by the rapid onset of symptoms that reflect the affected spinal cord segment [6]. These symptoms include, but are not limited to, back pain [7], sensory or motor deficits with different degrees of severity [5,8], bladder, bowel, or autonomic system dysfunction [3,9,10]. Moreover, rapid neurological deterioration is not uncommon and often suggestive of a worse prognosis [5,11].

SCInf may occur spontaneously or in a periprocedural or traumatic setting [11]. Spontaneous SCInf is defined as an ischemic event occurring without any identifiable inciting event, while periprocedural SCInf are the result of an iatrogenic or traumatic disruption of blood flow through spinal arteries originating in the aorta [12–15]. Regardless of the etiology, there are no definitive diagnostic criteria for SCInf. To address this gap, Zalewski et al. proposed a diagnostic algorithm that relies on clinical presentation, imaging, and other complementary diagnostic methods [11]. SCInf were divided into either spontaneous or periprocedural, and subclassified, based on the specificity of the diagnostic findings into definite, probable, or possible (Figure 1).

Spontaneous SCInf has been hypothesized to result from vascular disease processes analogous to those in cerebral stroke. However, the pathophysiology of spontaneous SCInf has been the subject of significantly less research [16]. Nonetheless, the presence of vascular risk factors such as hypertension, hyperlipidemia, diabetes, and smoking is well documented in patients with spontaneous SCInf [11]. However, more research is needed to clarify causal relationships.

While it is easier to diagnose periprocedural SCInf, as they are well-established complications mainly occurring in conjunction with aortic surgery, the diagnosis of spontaneous SCInf, remains more challenging. This complexity derives from several factors, of which the rarity of the condition [1–4], its overlap with other acute myelopathies [5,9,11], and the variability in presenting signs and symptoms, are the largest contributors. Additionally, previous studies seem to indicate that up to half of imaging workups in patients with a clinical suspicion of spinal cord infarction may return normal [17] and that a spinal

For peer review only - http://bmjopen.bmj.com/site/about/guidelines.xhtml

cord lesion may not be accurately discernible with T2-weighted imaging during the first 15 hours [13,15,18]. Hence, spontaneous SCInfs require a certain degree of clinical suspicion and expertise in order for a timely diagnosis to be made [15].

Currently, there is no clear guidelines for the management of SCInf [14]. Measures have been suggested, to prevent the occurrence of periprocedural ones. Although still a matter of debate [14,19–21], CSF drainage was proposed as a means to reduce intraspinal pressure and thereby enhance perfusion of the spinal cord [20]. In fact, recent reports addressing this topic have emphasized the value of maintaining an adequate spinal cord perfusion pressure, both during and after aortic procedures [22]. In contrast, the management of spontaneous SCInf, mainly relies on the treatment guidelines of ischemic cerebral stroke and myocardial infarction. Consequently, the mainstay consists of the reduction of cardiovascular risk factors [23] and antiplatelet therapy in eligible patients [24]. In the acute phase, thrombolytic therapy has been described [23,25,26]. However, the available literature includes only a few cases and consequently evidence to support this treatment strategy is currently lacking. Similarly, the use of corticosteroids early in the course of SCInf have been suggested, but supporting evidence is scarce [27].

The ability to walk is an essential parameter in the aftermath of spinal cord injury. In SCInf, previous surveys are conflicting regarding the proportion of patients with preserved ambulatory function (walking with or without aids) ranging from 38% [9] to 70% [17]. Considering neurological function at long-term follow-up, previous studies seem to agree that a gradual improvement occurs during an extended period of time after SCInf [28,29]. Analysis of the predictors of unfavorable outcomes revealed multi-segment lesions [4], lower admission ASIA scores [17], and older age [2] as predictors of outcome. Sex has not been identified as an outcome predictor [17]. Although not consistent through the literature [29], there seems to be preliminary evidence pointing towards worse outcomes in patients with periprocedural SCInf, in contrast to those with spontaneous SCInf [2,30].

The lack of definitive guidelines for the diagnosis and management of SCInf, as well as the conflicting evidence present throughout the literature warrants a review to identify and summarize the current knowledge regarding risk factors, diagnosis, management , and outcomes of spontaneous spinal cord infarctions. The planned systematic review aims to synthesize the relevant knowledge pertaining to this topic as well as to highlight knowledge

For peer review only

gaps in need of intensified research efforts. Contrary to periprocedural SCInf where comprehensive systematic reviews have been extensively carried out [22,31–34], there are to the best of our knowledge none addressing spontaneous SCInf. Instead of the more classic PICO criteria (Population, Intervention, Comparison, Outcome), we decide to use the SPIDER criteria [35] (Sample, Phenomenon of Interest, Design, Evaluation, Research type) which we believe are better suited to the purpose of this review (Table 1).

Table 1. SPIDER criteria

Sample	Any patient
Phenomenon of Interest	Spontaneous and idiopathic spinal cord infarction
Design	Descriptive studies with numeric data
Evaluation	Epidemiology, risk factors, treatment, and outcomes
Research Type	Experimental and observational studies

Methods and analysis

Study registration

This protocol for an intended systematic review is reported according to the Preferred Reporting Items for Systematic Reviews and Meta-Analyses Protocol (PRISMA-P) statement of 2015 [36]. The PRISMA-P checklist is provided as supplementary material (Supplementary file 1). The systematic review protocol will also be registered on PROSPERO.

Patient and public involvement:

Patients were not involved in the design or conception of the study.

Eligibility criteria

Inclusion criteria

Type of studies

For peer review only - http://bmjopen.bmj.com/site/about/guidelines.xhtml

All peer reviewed and original studies, written in English and available in the PubMed, Embase, or Web of Science databases from inception and onwards, will be eligible for inclusion.

Type of participant

All patient with spontaneous and idiopathic spinal cord infarctions will be included, regardless of age, ethnicity, and sex.

Type of outcome measurements

Epidemiological data such as age, sex and socioeconomic factors, risk factors, diagnosis and management strategies, outcomes and predictors will all be addressed. Furthermore, outcome parameters including pathological mechanisms, quality of life, and mortality, will be explored upon sufficient data.

Exclusion criteria

Non-original publications such as reviews, editorials, and letters to the editor will be disregarded together with conference abstracts and case reports. Studies found in languages other than English will be excluded for practical reasons. Non-idiopathic cases of SCInf occurring after clear inciting events, such as surgery, trauma, or hypovolemic shock will be disregarded and excluded from the analysis. Studies only addressing SCInfs secondary to vertebral artery dissections will also be excluded, as this topic has specifically been addressed in a previous systematic review [37].

Databases and search strategy

An electronic database search will be performed in PubMed, Embase, and Web of Science. A filter will be used to exclude non-English studies. To illustrate the process, the preliminary search strategy for each of the databases is provided (Supplementary file 2).

Study selection

For peer review only

The records retrieved from the different databases will be exported into the Rayyan Software [38]. After deduplication, the records will be screened based on title and abstract by one reviewer, to eliminate records that are plainly irrelevant. This is necessary as an unmanageable number of records is foreseen due to the broad search strategy that will be used. In the next step, three independent and blinded reviewers will be assigned the task of examining the remaining records applying the eligibility criteria based on full-text reading. Potential disagreements will be resolved by discussion with a fourth reviewer. Finally, reference lists of the selected articles will be thoroughly reviewed for any potentially eligible studies that were previously missed. The whole process will be illustrated in a PRISMA flowchart which will be provided in the final manuscript.

<u>Data extraction</u>

Data from selected records will be extracted using a predefined extraction template, preliminarily including (1) general information—title, first author, journal, publication year, etc.; (2) study characteristics—study type, sample size, follow-up time, etc.; (3) patient characteristics and epidemiology—age, sex, spinal segment involved, presenting symptoms and neurologic function etc.; (4) diagnosis and treatment characteristics—diagnostic modalities, treatment strategy, etc.; and (5) outcomes—neurological outcomes, predictors of outcome, quality of life, etc. The collaboration of multiple reviewers will be sought to achieve thorough extraction of the data. The final work will be assessed and cross-checked to prevent any error.

<u>Assessment of risk of bias</u>

The Oxford Center for Evidence-Based Medicine system [39], modified by Wright et al, will be used to assess evidence levels [40,41] (table 2). The selected articles will be allocated to one of four levels based on methodological quality, since the fifth level (V) pertains to expert opinions which are systematically excluded from our study. Risk of bias will be assessed using the appropriate tools specific to the type of study, as defined by Ma et al [42].

For peer review only

Table 2. Level of evidence based on primary research question by Wright et al [40].

	Therapeutic Studies—Investigating the results of treatment	Prognostic Studies—Investigating the outcome of disease	Diagnostic Studies—Investigating a diagnostic test
Level I	1. Good-quality randomized controlled trial,<br>2. Systematic review of Level-I studies	1. Prospective study,<br>2. Systematic review of Level-I studies	1. Testing of previously developed diagnostic criteria in series of consecutive patients (with universally applied reference "gold" standard),<br>2. Systematic review of Level-I studies
Level II	1. Prospective cohort study,<br>2. Poor-quality randomized controlled trial,<br>3. Systematic review,<br>a. Level-II studies,<br>b. Nonhomogeneous Level-I studies	1. Retrospective study,<br>2. Study of untreated controls from a previous randomized controlled trial,<br>3. Systematic review of Level-II studies	1. Development of diagnostic criteria on basis of consecutive patients (with universally applied reference "gold" standard),<br>2. Systematic review of Level-II studies
Level III	1. Case-control study,<br>2. Retrospective cohort study,		1. Study of nonconsecutive patients (no consistently applied

For peer review only

	3. Systematic review of Level-III studies		reference "gold" standard),<br>2. Systematic review of Level-III studies
Level IV	Case series (with no, or historical, control group)	Case series	1. Case-control study,<br>2. Poor reference standard
Level V	Expert opinion	Expert opinion	Expert opinion

For peer review only

Data synthesis:

After extraction, the data obtained from eligible studies will be systematically presented. Topics of interest to this review are chosen as follows:

1. Patient characteristics: epidemiology and risk factors
2. SCInf characteristics: spinal cord level affected and associated presenting symptoms
3. Diagnosis: modalities used
4. Management: Treatment options and strategies adopted
5. Patient outcomes: neurological outcomes, and predictors, quality of life and mortality.

The synthesis will address each of the mentioned topics in separate sections. In the absence of a satisfactory amount or quality of data, the synthesis will primarily take a narrative form and pool the available evidence to achieve higher power and more reliable information. Meta-analysis will only be performed for research questions where rigorous, homogenous, and sufficient data is available. A similar methodology has been described in several of our previous works [43,44].

Ethics and dissemination

Ethics approval is not required for this systematic review as it is based on existing publications. We plan to submit our work to a peer-reviewed journal where the results will be openly available.

Competing interests

No competing interests are reported by any of the authors.

Author Contributions

- Victor Gabriel El-Hajj: conception & design of the work, drafting of the article, critical revision, and final approval of the version to be published.

For peer review only - http://bmjopen.bmj.com/site/about/guidelines.xhtml

- Vasilios Stenimahitis: conception & design of the work, drafting of the article, and final approval of the version to be published.
- Maria Gharios: conception & design of the work, drafting of the article, and final approval of the version to be published.
- Omar Ali Mahdi: conception & design of the work, drafting of the article, and final approval of the version to be published.
- Adrian Elmi-Terander: guarantor of the review, conception & design of the work, drafting of the article, critical revision, and final approval of the version to be published.
- Erik Edström: conception & design of the work, drafting of the article, critical revision, and final approval of the version to be published.

Funding

AET was supported by Region Stockholm (clinical research appointment). None of the other authors received funding.

Ethics declarations

Ethics approval and consent to participate

Not applicable.

Consent for publication

Not applicable.

Acknowledgements

Not applicable.

For peer review only

References

1 Masson C, Pruvo JP, Meder JF, *et al.* Spinal cord infarction: clinical and magnetic resonance imaging findings and short term outcome. *J Neurol Neurosurg Psychiatry* 2004;**75**:1431–5. doi:10.1136/JNNP.2003.031724

2 Salvador de la Barrera S, Barca-Buyo A, Montoto-Marqués A, *et al.* Spinal cord infarction: prognosis and recovery in a series of 36 patients. *Spinal Cord* 2001;**39**:520–5. doi:10.1038/SJ.SC.3101201

3 Yadav N, Pendharkar H, Kulkarni GB. Spinal Cord Infarction: Clinical and Radiological Features. *J Stroke Cerebrovasc Dis* 2018;**27**:2810–21. doi:10.1016/J.JSTROKECEREBROVASDIS.2018.06.008

4 Hsu JL, Cheng MY, Liao MF, *et al.* The etiologies and prognosis associated with spinal cord infarction. *Ann Clin Transl Neurol* 2019;**6**:1456–64. doi:10.1002/ACN3.50840

5 Vargas MI, Gariani J, Sztajzel R, *et al.* Spinal cord ischemia: practical imaging tips, pearls, and pitfalls. *AJNR Am J Neuroradiol* 2015;**36**:825–30. doi:10.3174/AJNR.A4118

6 Romi F, Naess H. Spinal Cord Infarction in Clinical Neurology: A Review of Characteristics and Long-Term Prognosis in Comparison to Cerebral Infarction. *Eur Neurol* 2016;**76**:95–8. doi:10.1159/000446700

7 Novy J, Carruzzo A, Maeder P, *et al.* Spinal cord ischemia: clinical and imaging patterns, pathogenesis, and outcomes in 27 patients. *Arch Neurol* 2006;**63**:1113–20. doi:10.1001/ARCHNEUR.63.8.1113

8 Vongveeranonchai N, Zawahreh M, Strbian D, *et al.* Evaluation of a patient with spinal cord infarction after a hypotensive episode. *Stroke* 2014;**45**:e203–5. doi:10.1161/STROKEAHA.114.006490

9 Cheshire WP, Santos CC, Massey EW, *et al.* Spinal cord infarction: etiology and outcome. *Neurology* 1996;**47**:321–30. doi:10.1212/WNL.47.2.321

10 Cheung AT, Weiss SJ, McGarvey ML, *et al.* Interventions for reversing delayed-onset postoperative paraplegia after thoracic aortic reconstruction. *Ann Thorac Surg* 2002;**74**:413–21. doi:10.1016/S0003-4975(02)03714-1

11 Zalewski NL, Rabinstein AA, Krecke KN, *et al.* Characteristics of Spontaneous Spinal Cord Infarction and Proposed Diagnostic Criteria. *JAMA Neurol* 2019;**76**:56–63. doi:10.1001/JAMANEUROL.2018.2734

12 Lin WP, Kuan TS, Lin CI, *et al.* Spinal cord infarction during physical exertion due to polycythemia vera and aortoiliac occlusive disease: A case report. *Medicine* 2018;**97**. doi:10.1097/MD.0000000000012181

13 Pikija S, Kunz AB, Nardone R, *et al.* Spontaneous spinal cord infarction in Austria: a two-center comparative study. *Ther Adv Neurol Disord* 2022;**15**. doi:10.1177/17562864221076321

14 Ros Castelló V, Sánchez Sánchez A, Natera Villalba E, *et al.* Spinal cord infarction: aetiology, imaging findings, and prognostic factors in a series of 41 patients. *Neurologia* Published Online First: June 2021. doi:10.1016/J.NRLENG.2020.11.004

For peer review only

15 Pikija S, Mutzenbach JS, Kunz AB, *et al.* Delayed Hospital Presentation and Neuroimaging in Non-surgical Spinal Cord Infarction. *Front Neurol* 2017;**8**:143. doi:10.3389/FNEUR.2017.00143

16 Tubbs RS, Blouir MC, Romeo AK, *et al.* Spinal cord ischemia and atherosclerosis: a review of the literature. *Br J Neurosurg* 2011;**25**:666–70. doi:10.3109/02688697.2011.578774

17 Nedeltchev K, Loher TJ, Stepper F, *et al.* Long-term outcome of acute spinal cord ischemia syndrome. *Stroke* 2004;**35**:560–5. doi:10.1161/01.STR.0000111598.78198.EC

18 Weidauer S, Nichtweiß M, Hattingen E, *et al.* Spinal cord ischemia: aetiology, clinical syndromes and imaging features. *Neuroradiology* 2015;**57**:241–57. doi:10.1007/S00234-014-1464-6

19 Kramer CL. Vascular Disorders of the Spinal Cord. *Continuum (Minneap Minn)* 2018;**24**:407–26. doi:10.1212/CON.0000000000000595

20 Epstein N. Cerebrospinal fluid drains reduce risk of spinal cord injury for thoracic/thoracoabdominal aneurysm surgery: A review. *Surg Neurol Int* 2018;**9**. doi:10.4103/SNI.SNI_433_17

21 Visagan R, Hogg FRA, Gallagher MJ, *et al.* Monitoring Spinal Cord Tissue Oxygen in Patients With Acute, Severe Traumatic Spinal Cord Injuries. *Crit Care Med* 2022;**50**:E477–86. doi:10.1097/CCM.0000000000005433

22 Lella SK, Waller HD, Pendleton A, *et al.* A systematic review of spinal cord ischemia prevention and management after open and endovascular aortic repair. *J Vasc Surg* 2022;**75**:1091–106. doi:10.1016/J.JVS.2021.10.039

23 Haynes J, Shapiro M, Raz E, *et al.* Intra-arterial thrombolytic therapy for acute anterior spinal artery stroke. *J Clin Neurosci* 2021;**84**:102–5. doi:10.1016/J.JOCN.2020.11.035

24 Lynch K, Oster J, Apetauerova D, *et al.* Spinal cord stroke: acute imaging and intervention. *Case Rep Neurol Med* 2012;**2012**:1–3. doi:10.1155/2012/706780

25 Müller KI, Steffensen LH, Johnsen SH. Thrombolysis in anterior spinal artery syndrome. *BMJ Case Rep* 2012;**2012**. doi:10.1136/BCR-2012-006862

26 Jankovic J, Rey Bataillard V, Mercier N, *et al.* Acute ischemic myelopathy treated with intravenous thrombolysis: Four new cases and literature review. *Int J Stroke* 2019;**14**:893–7. doi:10.1177/1747493019851289

27 Robertson CS, Folitz R, Groosman RG, *et al.* Protection against experimental ischemic spinal cord injury. *J Neurosurg* 1986;**64**:633–42. doi:10.3171/JNS.1986.64.4.0633

28 Hanson SR, Romi F, Rekand T, *et al.* Long-term outcome after spinal cord infarctions. *Acta Neurol Scand* 2015;**131**:253–7. doi:10.1111/ANE.12343

29 Robertson CE, Brown RD, Wijdicks EFM, *et al.* Recovery after spinal cord infarcts: long-term outcome in 115 patients. *Neurology* 2012;**78**:114–21. doi:10.1212/WNL.0B013E31823EFC93

30 Foo D, Rossier AB. Anterior spinal artery syndrome and its natural history. *Paraplegia* 1983;**21**:1–10. doi:10.1038/SC.1983.1

31 de Haan P, Kalkman CJ, Jacobs MJHM. Pharmacologic neuroprotection in experimental spinal cord ischemia: a systematic review. *J Neurosurg Anesthesiol* 2001;**13**:3–12. doi:10.1097/00008506-200101000-00002

For peer review only - http://bmjopen.bmj.com/site/about/guidelines.xhtml

32 Choong AMTL, Wee IJY, Almond M, *et al.* A Systematic Review of the Use of Biochemical Markers in the Assessment of Spinal Cord Ischemia in Thoracoabdominal Aortic Aneurysm Repair. *https://doi.org/101177/1538574418814612* 2018;**53**:230–41. doi:10.1177/1538574418814612

33 Moulakakis KG, Alexiou VG, Karaolanis G, *et al.* Spinal Cord Ischemia following Elective Endovascular Repair of Infrarenal Aortic Aneurysms: A Systematic Review. *Ann Vasc Surg* 2018;**52**:280–91. doi:10.1016/j.avsg.2018.03.042

34 Zhang Z, Zhou Y, Lin S, *et al.* Systematic review and meta-analysis of association of prophylactic cerebrospinal fluid drainage in preventing spinal cord ischemia after thoracic endovascular aortic repair. *J Vasc Surg* 2022;**75**:1478-1489.e5. doi:10.1016/j.jvs.2021.10.050

35 Cooke A, Smith D, Booth A. Beyond PICO: The SPIDER tool for qualitative evidence synthesis. *Qual Health Res* 2012;**22**:1435–43. doi:10.1177/1049732312452938

36 Moher D, Shamseer L, Clarke M, *et al.* Preferred reporting items for systematic review and meta-analysis protocols (PRISMA-P) 2015 statement. *Revista Espanola de Nutricion Humana y Dietetica* 2016;**20**:148–60. doi:10.1186/2046-4053-4-1/TABLES/4

37 Hsu CY, Cheng CY, Lee J der, *et al.* Clinical features and outcomes of spinal cord infarction following vertebral artery dissection: a systematic review of the literature. *http://dx.doi.org/101179/1743132813Y0000000183* 2013;**35**:676–83. doi:10.1179/1743132813Y.0000000183

38 Ouzzani M, Hammady H, Fedorowicz Z, *et al.* Rayyan—a web and mobile app for systematic reviews. *Syst Rev* 2016;**5**:210. doi:10.1186/s13643-016-0384-4

39 OCEBM Levels of Evidence Working Group*. 'The Oxford 2011 Levels of Evidence'. Oxford Centre for Evidence-Based Medicine. http://www.cebm.net/index.aspx?o=5653 * OCEBM Table of Evidence Working Group = Jeremy Howick, Iain Chalmers (James Lind Library), Paul Glasziou, Trish Greenhalgh, Carl Heneghan, Alessandro Liberati, Ivan Moschetti, Bob Phillips, Hazel Thornton, Olive Goddard and Mary Hodgkinson.

40 Wright JG, Swiontkowski MF, Heckman JD. Introducing levels of evidence to the journal. *J Bone Joint Surg Am* 2003;**85**:1–3.

41 Anderson KK, Tetreault L, Shamji MF, *et al.* Optimal Timing of Surgical Decompression for Acute Traumatic Central Cord Syndrome. *Neurosurgery* 2015;**77**:S15–32. doi:10.1227/NEU.0000000000000946

42 Ma LL, Wang YY, Yang ZH, *et al.* Methodological quality (risk of bias) assessment tools for primary and secondary medical studies: What are they and which is better? *Mil Med Res* 2020;**7**:1–11. doi:10.1186/S40779-020-00238-8/TABLES/1

43 El-Hajj VG, Pettersson Segerlind J, Burström G, *et al.* Current knowledge on spinal meningiomas: a systematic review protocol. *BMJ Open* 2022;**12**:e061614. doi:10.1136/BMJOPEN-2022-061614

44 Iop A, Gabriel El-Hajj V, Gharios M, *et al.* Extended Reality in Neurosurgical Education: A Systematic Review. *Sensors 2022, Vol 22, Page 6067* 2022;**22**:6067. doi:10.3390/S22166067

For peer review only

Figure 1. Flowchart illustrating the categorization of SCInf based on the certainty of diagnostic findings, as defined by Zalewski et al.

For peer review only

For peer review only - http://bmjopen.bmj.com/site/about/guidelines.xhtml

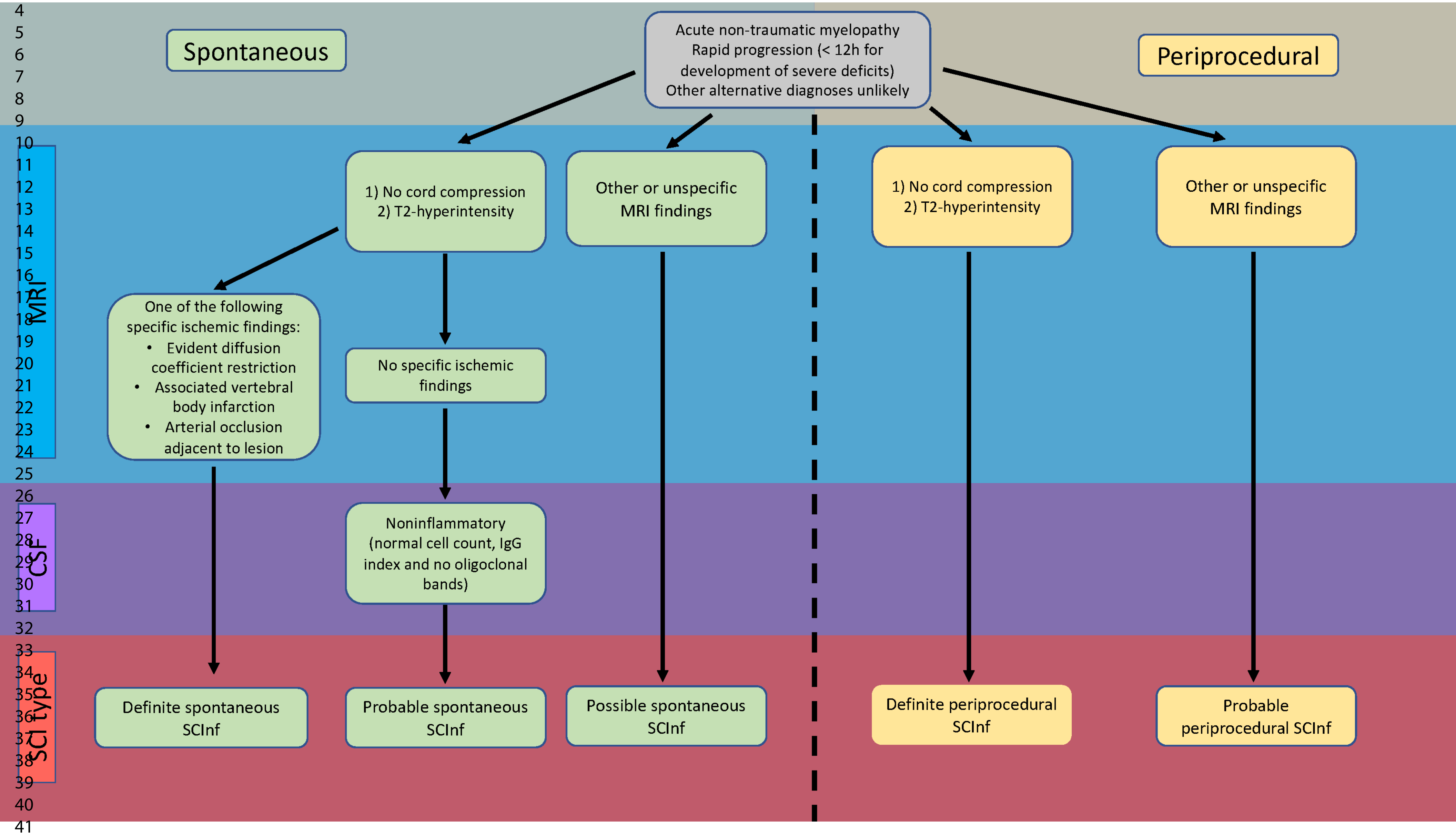
Spontaneous
Acute non-traumatic myelopathy
Rapid progression (< 12h for development of severe deficits)
Other alternative diagnoses unlikely
Periprocedural
MRI
CSF
SCI type
1) No cord compression
2) T2-hyperintensity
Other or unspecific MRI findings
1) No cord compression
2) T2-hyperintensity
Other or unspecific MRI findings
One of the following specific ischemic findings:
• Evident diffusion coefficient restriction
• Associated vertebral body infarction
• Arterial occlusion adjacent to lesion
No specific ischemic findings
Noninflammatory (normal cell count, IgG index and no oligoclonal bands)
Definite spontaneous SCInf
Probable spontaneous SCInf
Possible spontaneous SCInf
Definite periprocedural SCInf
Probable periprocedural SCInf

Reporting checklist for protocol of a systematic review and meta analysis.

Based on the PRISMA-P guidelines.

Reference: Moher D, Shamseer L, Clarke M, Ghersi D, Liberati A, Petticrew M, Shekelle P, Stewart LA. Preferred Reporting Items for Systematic Review and Meta-Analysis Protocols (PRISMA-P) 2015 statement. Syst Rev. 2015;4(1):1.

		Reporting Item	Section
**Title**			
Identification	#1a	Identify the report as a protocol of a systematic review	Title
Update	#1b	If the protocol is for an update of a previous systematic review, identify as such	n/a
**Registration**			
	#2	If registered, provide the name of the registry (such as PROSPERO) and registration number	This protocol will be registered on PROSPERO in due time
**Authors**			
Contact	#3a	Provide name, institutional affiliation, e-mail address of all protocol authors; provide physical mailing address of corresponding author	Author information
Contribution	#3b	Describe contributions of protocol authors and identify the guarantor of the review	Contributions
**Amendments**			
	#4	If the protocol represents an amendment of a previously completed or published protocol,	n/a

For peer review only

		identify as such and list changes; otherwise, state plan for documenting important protocol amendments	
**Support**			
Sources	#5a	Indicate sources of financial or other support for the review	n/a
Sponsor	#5b	Provide name for the review funder and / or sponsor	n/a
Role of sponsor or funder	#5c	Describe roles of funder(s), sponsor(s), and / or institution(s), if any, in developing the protocol	n/a
**Introduction**			
Rationale	#6	Describe the rationale for the review in the context of what is already known	Abstract and last part of Introduction
Objectives	#7	Provide an explicit statement of the question(s) the review will address with reference to participants, interventions, comparators, and outcomes (PICO)	Last part of introduction
**Methods**			
Eligibility criteria	#8	Specify the study characteristics (such as PICO, study design, setting, time frame) and report characteristics (such as years considered, language, publication status) to be used as criteria for eligibility for the review	Methods and analysis: Eligibility criteria section
Information sources	#9	Describe all intended information sources (such as electronic databases, contact with study authors, trial registers or other grey literature sources) with planned dates of coverage	Methods and analysis: Databases and search strategy section
Search strategy	#10	Present draft of search strategy to be used for at least one electronic database, including planned limits, such that it could be repeated	Methods and analysis: Databases and search strategy section and

For peer review only

			supplementary file 2
Study records - data management	#11a	Describe the mechanism(s) that will be used to manage records and data throughout the review	Methods and analysis: Study selection section
Study records - selection process	#11b	State the process that will be used for selecting studies (such as two independent reviewers) through each phase of the review (that is, screening, eligibility and inclusion in meta-analysis)	Methods and analysis: Study selection section
Study records - data collection process	#11c	Describe planned method of extracting data from reports (such as piloting forms, done independently, in duplicate), any processes for obtaining and confirming data from investigators	Methods and analysis: Data extraction section
Data items	#12	List and define all variables for which data will be sought (such as PICO items, funding sources), any pre-planned data assumptions and simplifications	Methods and analysis: Data extraction section
Outcomes and prioritization	#13	List and define all outcomes for which data will be sought, including prioritization of main and additional outcomes, with rationale	Methods and analysis: Data synthesis section
Risk of bias in individual studies	#14	Describe anticipated methods for assessing risk of bias of individual studies, including whether this will be done at the outcome or study level, or both; state how this information will be used in data synthesis	Methods and analysis: Risk of bias assessment and Quality of evidence sections
Data synthesis	#15a	Describe criteria under which study data will be quantitatively synthesised	n/a (only qualitative synthesis will be sought due to expected heterogeneity)
Data synthesis	#15b	If data are appropriate for quantitative synthesis, describe planned summary measures, methods of handling data and	n/a (qualitative synthesis only)

For peer review only

		methods of combining data from studies, including any planned exploration of consistency (such as I2, Kendall's τ)	
Data synthesis	#15c	Describe any proposed additional analyses (such as sensitivity or subgroup analyses, meta-regression)	n/a
Data synthesis	#15d	If quantitative synthesis is not appropriate, describe the type of summary planned	Methods and analysis: Data synthesis section
Meta-bias(es)	#16	Specify any planned assessment of meta-bias(es) (such as publication bias across studies, selective reporting within studies)	Methods and analysis: Risk of bias section
Confidence in cumulative evidence	#17	Describe how the strength of the body of evidence will be assessed (such as GRADE)	Methods and analysis: Risk of bias and Quality of evidence sections

The PRISMA-P elaboration and explanation paper is distributed under the terms of the Creative Commons Attribution License CC-BY. This checklist was completed on 29. January 2022 using https://www.goodreports.org/, a tool made by the EQUATOR Network in collaboration with Penelope.ai

For peer review only

Supplementary file 2: planned search strategy

Database	Search strategy
PubMed	((idiopathic OR spontaneous OR "non-surgical" OR "nonsurgical" OR "non-iatrogenic" OR "non iatrogenic" OR "noniatrogenic" OR "non-traumatic" OR "nontraumatic") AND ("spinal cord isch*" OR "spinal cord infarct*" OR "spinal isch*" OR "spinal infarct*")) OR ("spinal cord stroke" OR "spinal stroke" OR ”spinal artery infarct*” OR ”spinal artery isch*”) Filters: English
Web of Science	((idiopathic OR spontaneous OR "non-surgical" OR "nonsurgical" OR "non-iatrogenic" OR "non iatrogenic" OR "noniatrogenic" OR "non-traumatic" OR "nontraumatic") AND ("spinal cord isch*" OR "spinal cord infarct*" OR "spinal isch*" OR "spinal infarct*")) OR ("spinal cord stroke" OR "spinal stroke" OR ”spinal artery infarct*” OR ”spinal artery isch*”) (All Fields) and English (Languages)
Embase	((idiopathic OR spontaneous OR 'non-surgical' OR 'nonsurgical' OR 'non-iatrogenic' OR 'non iatrogenic' OR 'noniatrogenic' OR 'non-traumatic' OR 'nontraumatic') AND ('spinal cord isch*' OR 'spinal cord infarct*' OR 'spinal isch*' OR 'spinal infarct*') OR 'spinal cord stroke' OR 'spinal stroke' OR 'spinal artery infarction' OR 'spinal artery ischemia' OR 'spinal artery ischaemia') AND ([article]/lim OR [article in press]/lim OR [data papers]/lim OR [erratum]/lim OR [review]/lim) AND [humans]/lim AND [english]/lim AND [clinical study]/lim

For peer review only

BMJ Open

Spontaneous spinal cord infarctions: a systematic review and pooled analysis protocol

Journal:	*BMJ Open*
Manuscript ID	bmjopen-2022-071044.R1
Article Type:	Protocol
Date Submitted by the Author:	19-Apr-2023
Complete List of Authors:	El-Hajj, Victor Gabriel; Karolinska University Hospital, Neurosurgery<br>Stenimahitis, Vasilios; Karolinska University Hospital, Department of Neurology; Karolinska Institutet, Department of Clinical Neuroscience<br>Gharios, Maria; Karolinska University Hospital, Department of Neurology<br>Mahdi, Omar Ali; Karolinska University Hospital, Department of Neurology<br>Elmi-Terander, Adrian; Löwenströmska Hospital,<br>Edström, Erik; Karolinska Institutet, Department of Clinical Neuroscience
**Primary Subject Heading**:	Neurology
Secondary Subject Heading:	Neurology, Research methods
Keywords:	Stroke < NEUROLOGY, Stroke medicine < INTERNAL MEDICINE, NEUROLOGY

SCHOLARONE™ Manuscripts

Title: Spontaneous spinal cord infarctions: a systematic review and pooled analysis protocol

Author information

Author notes

[1]Victor Gabriel El-Hajj* (victor.gababriel.elhajj@stud.ki.se)

[1,2]Vasilios Stenimahitis*, MD (vasilios.stenimahitis@ki.se)

[1]Maria Gharios, BS (mcg02@mail.aub.edu)

[1]Omar Ali Mahdi (omar.ali.mahdi@stud.ki.se)

[3]Adrian Elmi-Terander, MD, PhD (Adrian.elmi.terander@gmail.com)

[2]Erik Edström, MD, PhD (erik.edstrom.1@ki.se)

*Contributed equally

Affiliations

[1]Department of Neurology, Karolinska University Hospital, Stockholm, Sweden

[2]Department of Clinical Neuroscience, Karolinska Institutet, Stockholm, Sweden

[3]Stockholm Spine Center, Löwenströmska Hospital, Upplands-Väsby, Sweden

Corresponding author

Victor Gabriel El-Hajj

Address: Karolinska University Hospital, Department of Neurosurgery, Eugeniavägen 6, 4th floor, Solna, Stockholm 17164.

ORCID: https://orcid.org/0000-0001-9479-761X

Email: victor.gabriel.elhajj@stud.ki.se

Number: +46765889449

Keywords: Spinal cord infarction, Stroke, Spontaneous infarction, Spinal cord injury

Word count: 1850 (excluding title page, abstract, statements & references).

For peer review only - http://bmjopen.bmj.com/site/about/guidelines.xhtml

Abstract

Introduction

Spinal cord infarction (SCInf) is a rare ischemic event that manifests with acute neurologic deficits. It is typically classified as either spontaneous, defined as SCInf without any inciting event, or periprocedural, which typically occur in conjunction with vascular surgery with aortic manipulations. While periprocedural SCInf have recently been the subject of intensified research, especially focusing on the primary prevention of this complication, spontaneous SCInf remains less studied.

Methods and Analysis

Electronic databases including PubMed, Web of Science and Embase, will be searched using the keywords ”spinal cord”, “infarction”, “ischemia” and “spontaneous”. The search will be set to provide only English studies published from database inception. Editorials, letters, and reviews will also be excluded. Reference lists of relevant records will also be searched. Identified studies will be screened for inclusion, by one reviewer in the first step and then three in the next step to decrease the risk of bias. The synthesis will address several topics of interest including epidemiology, presentation, diagnostics, treatment strategies, outcomes, and predictors. The review aims to gather the body of evidence to summarize the current knowledge on spinal cord infarctions. This will lead to a better understanding of the condition, its risk factors, diagnosis, and management. Moreover, the review will also provide an understanding of the prognosis of patients with SCInf with respect to neurologic function, quality of life, and mortality. Finally, this overview of the literature will allow the identification of knowledge gaps to help guide future research efforts.

Ethics and dissemination

Ethics approval was not required for our review as it is based on existing publications. The final manuscript will be submitted to a peer-reviewed journal.

For peer review only

Strengths and limitations

- Our wide search strategy and limited set of exclusion criteria allow many studies to be included, ensuring adequate coverage of the topic and correct identification of knowledge gaps.
- By providing a comprehensive synthesis of the body of evidence, the data can form the basis for management guidelines and future research efforts.
- We suspect that the quality of data does not suffice to perform a meta-analysis, consequently limiting the level of evidence that can be attained.

For peer review only

For peer review only - http://bmjopen.bmj.com/site/about/guidelines.xhtml

Introduction

Spinal cord infarction (SCInf) is a rare ischemic occurrence representing only a fraction of all ischemic strokes (1%) [1–4] and approximately 6% of all acute myelopathies [5]. The presentation of SCInf is characterized by the rapid onset of symptoms that reflect the affected spinal cord segment [6]. These symptoms include, but are not limited to, back pain [7], sensory or motor deficits with different degrees of severity [5,8], bladder, bowel, or autonomic system dysfunction [3,9,10]. Moreover, rapid neurological deterioration is not uncommon and often suggestive of a worse prognosis [5,11].

SCInf may occur spontaneously or in a periprocedural or traumatic setting [11,12]. Spontaneous SCInf is defined as an ischemic event occurring without any identifiable inciting event, while periprocedural SCInf are the result of an iatrogenic or traumatic disruption of blood flow through spinal arteries originating in the aorta [13–16]. Regardless of the etiology, there are no definitive diagnostic criteria for SCInf. To address this gap, Zalewski et al. proposed a diagnostic algorithm that relies on clinical presentation, imaging, and other complementary diagnostic methods [11]. SCInf were divided into either spontaneous or periprocedural, and subclassified, based on the specificity of the diagnostic findings into definite, probable, or possible (Figure 1).

Spontaneous SCInf has been hypothesized to result from vascular disease processes analogous to those in cerebral stroke. However, the pathophysiology of spontaneous SCInf has been the subject of significantly less research [17]. Nonetheless, the presence of vascular risk factors such as hypertension, hyperlipidemia, diabetes, and smoking is well documented in patients with spontaneous SCInf [11]. However, more research is needed to clarify causal relationships.

While it is easier to diagnose periprocedural SCInf, as they are well-established complications mainly occurring in conjunction with aortic surgery, the diagnosis of spontaneous SCInf, remains more challenging. This complexity derives from several factors, of which the rarity of the condition [1–4], its overlap with other acute myelopathies [5,9,11], and the variability in presenting signs and symptoms, are the largest contributors. Additionally, previous studies seem to indicate that up to half of imaging workups in patients with a clinical suspicion of spinal cord infarction may return normal [18] and that a spinal

For peer review only - http://bmjopen.bmj.com/site/about/guidelines.xhtml

cord lesion may not be accurately discernible with T2-weighted imaging during the first 15 hours [14,16,19]. Hence, spontaneous SCInfs require a certain degree of clinical suspicion and expertise in order for a timely diagnosis to be made [16].

Currently, there is no clear guidelines for the management of SCInf [15]. Measures have been suggested, to prevent the occurrence of periprocedural ones. Although still a matter of debate [15,20–22], CSF drainage was proposed as a means to reduce intraspinal pressure and thereby enhance perfusion of the spinal cord [21]. In fact, recent reports addressing this topic have emphasized the value of maintaining an adequate spinal cord perfusion pressure, both during and after aortic procedures [23]. In contrast, the management of spontaneous SCInf, mainly relies on the treatment guidelines of ischemic cerebral stroke and myocardial infarction. Consequently, the mainstay consists of the reduction of cardiovascular risk factors [24] and antiplatelet therapy in eligible patients [25]. In the acute phase, thrombolytic therapy has been described [24,26,27]. However, the available literature includes only a few cases and consequently evidence to support this treatment strategy is currently lacking. Similarly, the use of corticosteroids early in the course of SCInf have been suggested, but supporting evidence is scarce [28].

The ability to walk is an essential parameter in the aftermath of spinal cord injury. In SCInf, previous surveys are conflicting regarding the proportion of patients with preserved ambulatory function (walking with or without aids) ranging from 38% [9] to 70% [18]. Considering neurological function at long-term follow-up, previous studies seem to agree that a gradual improvement occurs during an extended period of time after SCInf [29,30]. Analysis of the predictors of unfavorable outcomes revealed multi-segment lesions [4], lower admission ASIA scores [18], and older age [2] as predictors of outcome. Sex has not been identified as an outcome predictor [18]. Although not consistent through the literature [30], there seems to be preliminary evidence pointing towards worse outcomes in patients with periprocedural SCInf, in contrast to those with spontaneous SCInf [2,31].

The lack of definitive guidelines for the diagnosis and management of SCInf, as well as the conflicting evidence present throughout the literature warrants a review to identify and summarize the current knowledge regarding risk factors, diagnosis, management , and outcomes of spontaneous spinal cord infarctions. The planned systematic review aims to synthesize the relevant knowledge pertaining to this topic as well as to highlight knowledge

For peer review only

gaps in need of intensified research efforts. Contrary to periprocedural SCInf where comprehensive systematic reviews have been extensively carried out [23,32–35], there are to the best of our knowledge none addressing spontaneous SCInf. Instead of the more classic PICO criteria (Population, Intervention, Comparison, Outcome), we decide to use the SPIDER criteria [36] (Sample, Phenomenon of Interest, Design, Evaluation, Research type) which we believe are better suited to the purpose of this review (Table 1).

Table 1. SPIDER criteria

Sample	Any patient
Phenomenon of Interest	Spontaneous spinal cord infarction
Design	Descriptive studies with numeric data
Evaluation	Epidemiology, risk factors, treatment, and outcomes
Research Type	Experimental and observational studies

Methods and analysis

Study registration

This protocol for an intended systematic review is reported according to the Preferred Reporting Items for Systematic Reviews and Meta-Analyses Protocol (PRISMA-P) statement of 2015 [37]. The PRISMA-P checklist is provided as supplementary material (Supplementary file 1). The systematic review protocol was registered on PROSPERO (registration ID: CED42023393241; registration date: 24/02/2023).

Patient and public involvement:

Patients were not involved in the design or conception of the study.

Eligibility criteria

Inclusion criteria

Type of studies

For peer review only

All peer reviewed and original studies, written in English and available in the PubMed, Embase, or Web of Science databases from inception and onwards, will be eligible for inclusion.

Type of participant

All patient with spontaneous spinal cord infarctions will be included, regardless of age, ethnicity, and sex.

Type of outcome measurements

Epidemiological data such as age, sex and socioeconomic factors, risk factors, diagnosis and management strategies, outcomes and predictors will all be addressed. Furthermore, outcome parameters including pathological mechanisms, quality of life, and mortality, will be explored upon sufficient data.

Exclusion criteria

Non-original publications such as reviews, editorials, and letters to the editor will be disregarded together with conference abstracts and case reports. Studies found in languages other than English will be excluded for practical reasons. Non-spontaneous cases of SCInf occurring after clear inciting events, such as surgery, trauma, or hypovolemic shock will be disregarded and excluded from the analysis. Studies only addressing SCInfs secondary to vertebral artery dissections will also be excluded, as this topic has specifically been addressed in a previous systematic review [38].

<u>Databases and search strategy</u>

An electronic database search will be performed in PubMed, Embase, and Web of Science. A filter will be used to exclude non-English studies. To illustrate the process, the preliminary search strategy for each of the databases is provided (Supplementary file 2).

<u>Study selection</u>

For peer review only - http://bmjopen.bmj.com/site/about/guidelines.xhtml

The records retrieved from the different databases will be exported into the Rayyan Software [39]. After deduplication, the records will be screened based on title and abstract by one reviewer, to eliminate records that are plainly irrelevant. This is necessary as an unmanageable number of records is foreseen due to the broad search strategy that will be used. In the next step, three independent and blinded reviewers will be assigned the task of examining the remaining records applying the eligibility criteria based on full-text reading. Potential disagreements will be resolved by discussion with a fourth reviewer. Finally, reference lists of the selected articles will be thoroughly reviewed for any potentially eligible studies that were previously missed. The whole process will be illustrated in a PRISMA flowchart which will be provided in the final manuscript.

For peer review only

Data extraction

Data from selected records will be extracted using a predefined extraction template, preliminarily including (1) general information—title, first author, journal, publication year, etc.; (2) study characteristics—study type, sample size, follow-up time, etc.; (3) patient characteristics and epidemiology—age, sex, spinal segment involved, presenting symptoms and neurologic function etc.; (4) diagnosis and treatment characteristics—diagnostic modalities, treatment strategy, etc.; and (5) outcomes—neurological outcomes, predictors of outcome, quality of life, etc. The collaboration of multiple reviewers will be sought to achieve thorough extraction of the data. The final work will be assessed and cross-checked to prevent any error.

Assessment of risk of bias

The Oxford Center for Evidence-Based Medicine system [40], modified by Wright et al, will be used to assess evidence levels [41,42] (table 2). The selected articles will be allocated to one of four levels based on methodological quality, since the fifth level (V) pertains to expert opinions which are systematically excluded from our study. Risk of bias will be assessed using the appropriate tools specific to the type of study, as defined by Ma et al [43].

For peer review only - http://bmjopen.bmj.com/site/about/guidelines.xhtml

Table 2. Level of evidence based on primary research question by Wright et al [41].

	Therapeutic Studies—Investigating the results of treatment	Prognostic Studies—Investigating the outcome of disease	Diagnostic Studies—Investigating a diagnostic test
Level I	1. Good-quality randomized controlled trial,<br>2. Systematic review of Level-I studies	1. Prospective study,<br>2. Systematic review of Level-I studies	1. Testing of previously developed diagnostic criteria in series of consecutive patients (with universally applied reference "gold" standard),<br>2. Systematic review of Level-I studies
Level II	1. Prospective cohort study,<br>2. Poor-quality randomized controlled trial,<br>3. Systematic review,<br>a. Level-II studies,<br>b. Nonhomogeneous Level-I studies	1. Retrospective study,<br>2. Study of untreated controls from a previous randomized controlled trial,<br>3. Systematic review of Level-II studies	1. Development of diagnostic criteria on basis of consecutive patients (with universally applied reference "gold" standard),<br>2. Systematic review of Level-II studies
Level III	1. Case-control study,<br>2. Retrospective cohort study,		1. Study of nonconsecutive patients (no consistently applied

For peer review only

	3. Systematic review of Level-III studies		reference "gold" standard),<br>2. Systematic review of Level-III studies
Level IV	Case series (with no, or historical, control group)	Case series	1. Case-control study,<br>2. Poor reference standard
Level V	Expert opinion	Expert opinion	Expert opinion

For peer review only

For peer review only - http://bmjopen.bmj.com/site/about/guidelines.xhtml

Data synthesis:

After extraction, the data obtained from eligible studies will be systematically presented. Topics of interest to this review are chosen as follows:

1. Patient characteristics: epidemiology and risk factors
2. SCInf characteristics: spinal cord level affected and associated presenting symptoms
3. Diagnosis: modalities used
4. Management: Treatment options and strategies adopted
5. Patient outcomes: neurological outcomes, and predictors, quality of life and mortality.

The synthesis will address each of the mentioned topics in separate sections. In the absence of a satisfactory amount or quality of data, the synthesis will primarily take a narrative form and pool the available evidence to achieve higher power and more reliable information. Meta-analysis will only be performed for research questions where rigorous, homogenous, and sufficient data is available. A similar methodology has been described in several of our previous works [44–47].

Ethics and dissemination

Ethics approval is not required for this systematic review as it is based on existing publications. The review is planned to take place between the months of February and August of the year of 2023. We then plan to submit our work to a peer-reviewed journal where the results will be openly available.

Competing interests

No competing interests are reported by any of the authors.

Author Contributions

- Victor Gabriel El-Hajj: conception & design of the work, drafting of the article, critical revision, and final approval of the version to be published.

For peer review only - http://bmjopen.bmj.com/site/about/guidelines.xhtml

- Vasilios Stenimahitis: conception & design of the work, drafting of the article, and final approval of the version to be published.
- Maria Gharios: conception & design of the work, drafting of the article, and final approval of the version to be published.
- Omar Ali Mahdi: conception & design of the work, drafting of the article, and final approval of the version to be published.
- Adrian Elmi-Terander: guarantor of the review, conception & design of the work, drafting of the article, critical revision, and final approval of the version to be published.
- Erik Edström: conception & design of the work, drafting of the article, critical revision, and final approval of the version to be published.

Funding

AET was supported by Region Stockholm (clinical research appointment). None of the other authors received funding.

Ethics declarations

Ethics approval and consent to participate

Not applicable.

Consent for publication

Not applicable.

Acknowledgements

Not applicable.

For peer review only

References

1 Masson C, Pruvo JP, Meder JF, *et al.* Spinal cord infarction: clinical and magnetic resonance imaging findings and short term outcome. *J Neurol Neurosurg Psychiatry* 2004;**75**:1431–5. doi:10.1136/JNNP.2003.031724

2 Salvador de la Barrera S, Barca-Buyo A, Montoto-Marqués A, *et al.* Spinal cord infarction: prognosis and recovery in a series of 36 patients. *Spinal Cord* 2001;**39**:520–5. doi:10.1038/SJ.SC.3101201

3 Yadav N, Pendharkar H, Kulkarni GB. Spinal Cord Infarction: Clinical and Radiological Features. *J Stroke Cerebrovasc Dis* 2018;**27**:2810–21. doi:10.1016/J.JSTROKECEREBROVASDIS.2018.06.008

4 Hsu JL, Cheng MY, Liao MF, *et al.* The etiologies and prognosis associated with spinal cord infarction. *Ann Clin Transl Neurol* 2019;**6**:1456–64. doi:10.1002/ACN3.50840

5 Vargas MI, Gariani J, Sztajzel R, *et al.* Spinal cord ischemia: practical imaging tips, pearls, and pitfalls. *AJNR Am J Neuroradiol* 2015;**36**:825–30. doi:10.3174/AJNR.A4118

6 Romi F, Naess H. Spinal Cord Infarction in Clinical Neurology: A Review of Characteristics and Long-Term Prognosis in Comparison to Cerebral Infarction. *Eur Neurol* 2016;**76**:95–8. doi:10.1159/000446700

7 Novy J, Carruzzo A, Maeder P, *et al.* Spinal cord ischemia: clinical and imaging patterns, pathogenesis, and outcomes in 27 patients. *Arch Neurol* 2006;**63**:1113–20. doi:10.1001/ARCHNEUR.63.8.1113

8 Vongveeranonchai N, Zawahreh M, Strbian D, *et al.* Evaluation of a patient with spinal cord infarction after a hypotensive episode. *Stroke* 2014;**45**:e203–5. doi:10.1161/STROKEAHA.114.006490

9 Cheshire WP, Santos CC, Massey EW, *et al.* Spinal cord infarction: etiology and outcome. *Neurology* 1996;**47**:321–30. doi:10.1212/WNL.47.2.321

10 Cheung AT, Weiss SJ, McGarvey ML, *et al.* Interventions for reversing delayed-onset postoperative paraplegia after thoracic aortic reconstruction. *Ann Thorac Surg* 2002;**74**:413–21. doi:10.1016/S0003-4975(02)03714-1

11 Zalewski NL, Rabinstein AA, Krecke KN, *et al.* Characteristics of Spontaneous Spinal Cord Infarction and Proposed Diagnostic Criteria. *JAMA Neurol* 2019;**76**:56–63. doi:10.1001/JAMANEUROL.2018.2734

12 Stenimahitis V, Fletcher-Sandersjöö A, El-Hajj VG, *et al.* Long-term outcomes following periprocedural and spontaneous spinal cord infarctions: a population-based cohort study. *Neurology* 2023; **[In press]**.

13 Lin WP, Kuan TS, Lin CI, *et al.* Spinal cord infarction during physical exertion due to polycythemia vera and aortoiliac occlusive disease: A case report. *Medicine* 2018;**97**. doi:10.1097/MD.0000000000012181

14 Pikija S, Kunz AB, Nardone R, *et al.* Spontaneous spinal cord infarction in Austria: a two-center comparative study. *Ther Adv Neurol Disord* 2022;**15**. doi:10.1177/17562864221076321

For peer review only - http://bmjopen.bmj.com/site/about/guidelines.xhtml

15 Ros Castelló V, Sánchez Sánchez A, Natera Villalba E, *et al.* Spinal cord infarction: aetiology, imaging findings, and prognostic factors in a series of 41 patients. *Neurologia* Published Online First: June 2021. doi:10.1016/J.NRLENG.2020.11.004
16 Pikija S, Mutzenbach JS, Kunz AB, *et al.* Delayed Hospital Presentation and Neuroimaging in Non-surgical Spinal Cord Infarction. *Front Neurol* 2017;**8**:143. doi:10.3389/FNEUR.2017.00143
17 Tubbs RS, Blouir MC, Romeo AK, *et al.* Spinal cord ischemia and atherosclerosis: a review of the literature. *Br J Neurosurg* 2011;**25**:666–70. doi:10.3109/02688697.2011.578774
18 Nedeltchev K, Loher TJ, Stepper F, *et al.* Long-term outcome of acute spinal cord ischemia syndrome. *Stroke* 2004;**35**:560–5. doi:10.1161/01.STR.0000111598.78198.EC
19 Weidauer S, Nichtweiß M, Hattingen E, *et al.* Spinal cord ischemia: aetiology, clinical syndromes and imaging features. *Neuroradiology* 2015;**57**:241–57. doi:10.1007/S00234-014-1464-6
20 Kramer CL. Vascular Disorders of the Spinal Cord. *Continuum (Minneap Minn)* 2018;**24**:407–26. doi:10.1212/CON.0000000000000595
21 Epstein N. Cerebrospinal fluid drains reduce risk of spinal cord injury for thoracic/thoracoabdominal aneurysm surgery: A review. *Surg Neurol Int* 2018;**9**. doi:10.4103/SNI.SNI_433_17
22 Visagan R, Hogg FRA, Gallagher MJ, *et al.* Monitoring Spinal Cord Tissue Oxygen in Patients With Acute, Severe Traumatic Spinal Cord Injuries. *Crit Care Med* 2022;**50**:E477–86. doi:10.1097/CCM.0000000000005433
23 Lella SK, Waller HD, Pendleton A, *et al.* A systematic review of spinal cord ischemia prevention and management after open and endovascular aortic repair. *J Vasc Surg* 2022;**75**:1091–106. doi:10.1016/J.JVS.2021.10.039
24 Haynes J, Shapiro M, Raz E, *et al.* Intra-arterial thrombolytic therapy for acute anterior spinal artery stroke. *J Clin Neurosci* 2021;**84**:102–5. doi:10.1016/J.JOCN.2020.11.035
25 Lynch K, Oster J, Apetauerova D, *et al.* Spinal cord stroke: acute imaging and intervention. *Case Rep Neurol Med* 2012;**2012**:1–3. doi:10.1155/2012/706780
26 Müller KI, Steffensen LH, Johnsen SH. Thrombolysis in anterior spinal artery syndrome. *BMJ Case Rep* 2012;**2012**. doi:10.1136/BCR-2012-006862
27 Jankovic J, Rey Bataillard V, Mercier N, *et al.* Acute ischemic myelopathy treated with intravenous thrombolysis: Four new cases and literature review. *Int J Stroke* 2019;**14**:893–7. doi:10.1177/1747493019851289
28 Robertson CS, Folitz R, Groosman RG, *et al.* Protection against experimental ischemic spinal cord injury. *J Neurosurg* 1986;**64**:633–42. doi:10.3171/JNS.1986.64.4.0633
29 Hanson SR, Romi F, Rekand T, *et al.* Long-term outcome after spinal cord infarctions. *Acta Neurol Scand* 2015;**131**:253–7. doi:10.1111/ANE.12343
30 Robertson CE, Brown RD, Wijdicks EFM, *et al.* Recovery after spinal cord infarcts: long-term outcome in 115 patients. *Neurology* 2012;**78**:114–21. doi:10.1212/WNL.0B013E31823EFC93
31 Foo D, Rossier AB. Anterior spinal artery syndrome and its natural history. *Paraplegia* 1983;**21**:1–10. doi:10.1038/SC.1983.1

For peer review only - http://bmjopen.bmj.com/site/about/guidelines.xhtml

32 de Haan P, Kalkman CJ, Jacobs MJHM. Pharmacologic neuroprotection in experimental spinal cord ischemia: a systematic review. *J Neurosurg Anesthesiol* 2001;**13**:3–12. doi:10.1097/00008506-200101000-00002

33 Choong AMTL, Wee IJY, Almond M, *et al.* A Systematic Review of the Use of Biochemical Markers in the Assessment of Spinal Cord Ischemia in Thoracoabdominal Aortic Aneurysm Repair. *https://doi.org/101177/1538574418814612* 2018;**53**:230–41. doi:10.1177/1538574418814612

34 Moulakakis KG, Alexiou VG, Karaolanis G, *et al.* Spinal Cord Ischemia following Elective Endovascular Repair of Infrarenal Aortic Aneurysms: A Systematic Review. *Ann Vasc Surg* 2018;**52**:280–91. doi:10.1016/j.avsg.2018.03.042

35 Zhang Z, Zhou Y, Lin S, *et al.* Systematic review and meta-analysis of association of prophylactic cerebrospinal fluid drainage in preventing spinal cord ischemia after thoracic endovascular aortic repair. *J Vasc Surg* 2022;**75**:1478-1489.e5. doi:10.1016/j.jvs.2021.10.050

36 Cooke A, Smith D, Booth A. Beyond PICO: The SPIDER tool for qualitative evidence synthesis. *Qual Health Res* 2012;**22**:1435–43. doi:10.1177/1049732312452938

37 Moher D, Shamseer L, Clarke M, *et al.* Preferred reporting items for systematic review and meta-analysis protocols (PRISMA-P) 2015 statement. *Revista Espanola de Nutricion Humana y Dietetica* 2016;**20**:148–60. doi:10.1186/2046-4053-4-1/TABLES/4

38 Hsu CY, Cheng CY, Lee J der, *et al.* Clinical features and outcomes of spinal cord infarction following vertebral artery dissection: a systematic review of the literature. *http://dx.doi.org/101179/1743132813Y0000000183* 2013;**35**:676–83. doi:10.1179/1743132813Y.0000000183

39 Ouzzani M, Hammady H, Fedorowicz Z, *et al.* Rayyan—a web and mobile app for systematic reviews. *Syst Rev* 2016;**5**:210. doi:10.1186/s13643-016-0384-4

40 OCEBM Levels of Evidence Working Group*. 'The Oxford 2011 Levels of Evidence'. Oxford Centre for Evidence-Based Medicine. http://www.cebm.net/index.aspx?o=5653 * OCEBM Table of Evidence Working Group = Jeremy Howick, Iain Chalmers (James Lind Library), Paul Glasziou, Trish Greenhalgh, Carl Heneghan, Alessandro Liberati, Ivan Moschetti, Bob Phillips, Hazel Thornton, Olive Goddard and Mary Hodgkinson.

41 Wright JG, Swiontkowski MF, Heckman JD. Introducing levels of evidence to the journal. *J Bone Joint Surg Am* 2003;**85**:1–3.

42 Anderson KK, Tetreault L, Shamji MF, *et al.* Optimal Timing of Surgical Decompression for Acute Traumatic Central Cord Syndrome. *Neurosurgery* 2015;**77**:S15–32. doi:10.1227/NEU.0000000000000946

43 Ma LL, Wang YY, Yang ZH, *et al.* Methodological quality (risk of bias) assessment tools for primary and secondary medical studies: What are they and which is better? *Mil Med Res* 2020;**7**:1–11. doi:10.1186/S40779-020-00238-8/TABLES/1

44 El-Hajj VG, Pettersson Segerlind J, Burström G, *et al.* Current knowledge on spinal meningiomas: a systematic review protocol. *BMJ Open* 2022;**12**:e061614. doi:10.1136/BMJOPEN-2022-061614

For peer review only - http://bmjopen.bmj.com/site/about/guidelines.xhtml

45 El-Hajj VG, Pettersson-Segerlind J, Fletcher-Sandersjöö A, *et al.* Current Knowledge on Spinal Meningiomas Epidemiology, Tumor Characteristics and Non-Surgical Treatment Options: A Systematic Review and Pooled Analysis (Part 1). *Cancers (Basel)* 2022;**14**. doi:10.3390/CANCERS14246251
46 El-Hajj VG, Pettersson-Segerlind J, Fletcher-Sandersjöö A, *et al.* Current Knowledge on Spinal Meningiomas-Surgical Treatment, Complications, and Outcomes: A Systematic Review and Meta-Analysis (Part 2). *Cancers (Basel)* 2022;**14**. doi:10.3390/CANCERS14246221
47 Iop A, Gabriel El-Hajj V, Gharios M, *et al.* Extended Reality in Neurosurgical Education: A Systematic Review. *Sensors 2022, Vol 22, Page 6067* 2022;**22**:6067. doi:10.3390/S22166067

Figure 1. Flowchart illustrating the categorization of SCInf based on the certainty of diagnostic findings, as defined by Zalewski et al.

For peer review only

For peer review only - http://bmjopen.bmj.com/site/about/guidelines.xhtml

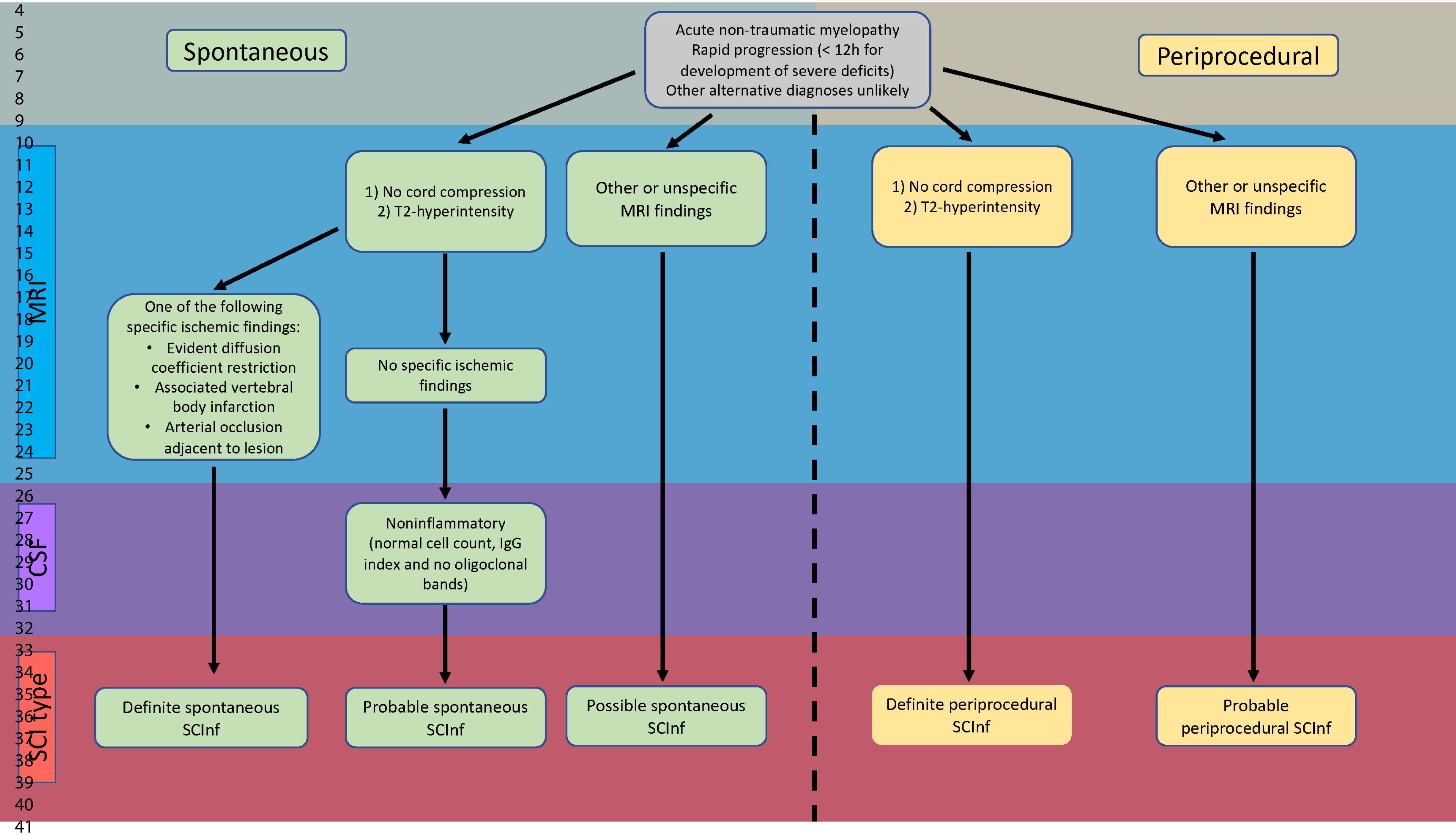
Spontaneous
Acute non-traumatic myelopathy
Rapid progression (< 12h for development of severe deficits)
Other alternative diagnoses unlikely
Periprocedural
MRI
1) No cord compression
2) T2-hyperintensity
Other or unspecific MRI findings
1) No cord compression
2) T2-hyperintensity
Other or unspecific MRI findings
One of the following specific ischemic findings:
• Evident diffusion coefficient restriction
• Associated vertebral body infarction
• Arterial occlusion adjacent to lesion
No specific ischemic findings
CSF
Noninflammatory (normal cell count, IgG index and no oligoclonal bands)
SCI type
Definite spontaneous SCInf
Probable spontaneous SCInf
Possible spontaneous SCInf
Definite periprocedural SCInf
Probable periprocedural SCInf

Reporting checklist for protocol of a systematic review and meta analysis.

Based on the PRISMA-P guidelines.

Reference: Moher D, Shamseer L, Clarke M, Ghersi D, Liberati A, Petticrew M, Shekelle P, Stewart LA. Preferred Reporting Items for Systematic Review and Meta-Analysis Protocols (PRISMA-P) 2015 statement. Syst Rev. 2015;4(1):1.

For peer review only

		Reporting Item	Section (page)
**Title**			
Identification	#1a	Identify the report as a protocol of a systematic review	Title (1)
Update	#1b	If the protocol is for an update of a previous systematic review, identify as such	n/a
**Registration**			
	#2	If registered, provide the name of the registry (such as PROSPERO) and registration number	Study registration (6)
**Authors**			
Contact	#3a	Provide name, institutional affiliation, e-mail address of all protocol authors; provide physical mailing address of corresponding author	Author information (1)
Contribution	#3b	Describe contributions of protocol authors and identify the guarantor of the review	Contributions (11-12)
**Amendments**			
	#4	If the protocol represents an amendment of a previously completed or published protocol,	n/a

		identify as such and list changes; otherwise, state plan for documenting important protocol amendments	
**Support**			
Sources	#5a	Indicate sources of financial or other support for the review	n/a
Sponsor	#5b	Provide name for the review funder and / or sponsor	n/a
Role of sponsor or funder	#5c	Describe roles of funder(s), sponsor(s), and / or institution(s), if any, in developing the protocol	n/a
**Introduction**			
Rationale	#6	Describe the rationale for the review in the context of what is already known	Abstract and last part of Introduction (2, and 5-6)
Objectives	#7	Provide an explicit statement of the question(s) the review will address with reference to participants, interventions, comparators, and outcomes (PICO)	Last part of introduction (5-6)
**Methods**			
Eligibility criteria	#8	Specify the study characteristics (such as PICO, study design, setting, time frame) and report characteristics (such as years considered, language, publication status) to be used as criteria for eligibility for the review	Methods and analysis: Eligibility criteria section (7)
Information sources	#9	Describe all intended information sources (such as electronic databases, contact with study authors, trial registers or other grey literature sources) with planned dates of coverage	Methods and analysis: Databases and search strategy section (7)
Search strategy	#10	Present draft of search strategy to be used for at least one electronic database, including planned limits, such that it could be repeated	Methods and analysis: Databases and search strategy section and

For peer review only

			supplementary file 2 (7)
Study records - data management	#11a	Describe the mechanism(s) that will be used to manage records and data throughout the review	Methods and analysis: Study selection section (8)
Study records - selection process	#11b	State the process that will be used for selecting studies (such as two independent reviewers) through each phase of the review (that is, screening, eligibility and inclusion in meta-analysis)	Methods and analysis: Study selection section (8)
Study records - data collection process	#11c	Describe planned method of extracting data from reports (such as piloting forms, done independently, in duplicate), any processes for obtaining and confirming data from investigators	Methods and analysis: Data extraction section (8)
Data items	#12	List and define all variables for which data will be sought (such as PICO items, funding sources), any pre-planned data assumptions and simplifications	Methods and analysis: Data extraction section (8)
Outcomes and prioritization	#13	List and define all outcomes for which data will be sought, including prioritization of main and additional outcomes, with rationale	Methods and analysis: Data synthesis section (11)
Risk of bias in individual studies	#14	Describe anticipated methods for assessing risk of bias of individual studies, including whether this will be done at the outcome or study level, or both; state how this information will be used in data synthesis	Methods and analysis: Risk of bias assessment and Quality of evidence sections (8-9)
Data synthesis	#15a	Describe criteria under which study data will be quantitatively synthesised	n/a (only qualitative synthesis will be sought due to expected heterogeneity)
Data synthesis	#15b	If data are appropriate for quantitative synthesis, describe planned summary measures, methods of handling data and	n/a (qualitative synthesis only)

For peer review only

		methods of combining data from studies, including any planned exploration of consistency (such as I2, Kendall's τ)	
Data synthesis	#15c	Describe any proposed additional analyses (such as sensitivity or subgroup analyses, meta-regression)	n/a
Data synthesis	#15d	If quantitative synthesis is not appropriate, describe the type of summary planned	Methods and analysis: Data synthesis section (11)
Meta-bias(es)	#16	Specify any planned assessment of meta-bias(es) (such as publication bias across studies, selective reporting within studies)	Methods and analysis: Risk of bias section (8-9)
Confidence in cumulative evidence	#17	Describe how the strength of the body of evidence will be assessed (such as GRADE)	Methods and analysis: Risk of bias and Quality of evidence sections (8-9)

The PRISMA-P elaboration and explanation paper is distributed under the terms of the Creative Commons Attribution License CC-BY. This checklist was completed on 29. January 2022 using https://www.goodreports.org/, a tool made by the EQUATOR Network in collaboration with Penelope.ai

For peer review only

Supplementary file 2: planned search strategy

**Database**	**Search strategy**
PubMed	((idiopathic OR spontaneous OR "non-surgical" OR "nonsurgical" OR "non-iatrogenic" OR "non iatrogenic" OR "noniatrogenic" OR "non-traumatic" OR "nontraumatic") AND ("spinal cord isch*" OR "spinal cord infarct*" OR "spinal isch*" OR "spinal infarct*")) OR ("spinal cord stroke" OR "spinal stroke" OR ”spinal artery infarct*” OR ”spinal artery isch*”) Filters: English
Web of Science	((idiopathic OR spontaneous OR "non-surgical" OR "nonsurgical" OR "non-iatrogenic" OR "non iatrogenic" OR "noniatrogenic" OR "non-traumatic" OR "nontraumatic") AND ("spinal cord isch*" OR "spinal infarct*" OR "spinal isch*" OR "spinal infarct*")) OR ("spinal cord stroke" OR "spinal stroke" OR ”spinal artery infarct*” OR ”spinal artery isch*”) (All Fields) and English (Languages)
Embase	((idiopathic OR spontaneous OR 'non-surgical' OR 'nonsurgical' OR 'non-iatrogenic' OR 'non iatrogenic' OR 'noniatrogenic' OR 'non-traumatic' OR 'nontraumatic') AND ('spinal cord isch*' OR 'spinal cord infarct*' OR 'spinal isch*' OR 'spinal infarct*') OR 'spinal cord stroke' OR 'spinal stroke' OR 'spinal artery infarction' OR 'spinal artery ischemia' OR 'spinal artery ischaemia') AND ([article]/lim OR [article in press]/lim OR [data papers]/lim OR [erratum]/lim OR [review]/lim) AND [humans]/lim AND [english]/lim AND [clinical study]/lim

For peer review only
